# Structure and function of the lysine methyltransferase SETD2 in cancer: From histones to cytoskeleton

**DOI:** 10.1016/j.neo.2024.101090

**Published:** 2024-11-25

**Authors:** Christina Michail, Fernando Rodrigues Lima, Mireille Viguier, Frédérique Deshayes

**Affiliations:** Université Paris Cité, CNRS, Unité de Biologie Fonctionnelle et Adaptative, F-75013 Paris, France

**Keywords:** Cancer, Cytoskeleton, Genetic alterations, Histones, SETD2, Structure

## Abstract

SETD2 is known to be the unique histone methyltransferase responsible for the trimethylation of the lysine 36 of histone H3 thus generating H3K36me3. This epigenetic mark is critical for transcriptional activation and elongation, DNA repair, mRNA splicing, and DNA methylation. Recurrent SETD2-inactivating mutations and altered H3K36me3 levels are found in cancer at high frequency and numerous studies indicate that SETD2 acts as a tumor suppressor. Recently, SETD2 was further shown to methylate non-histone proteins particularly the cytoskeletal proteins tubulin and actin with subsequent impacts on cytoskeleton structure, mitosis and cell migration.

Herein, we provide a review of the role of SETD2 in different cancers with special emphasis on the structural basis of the functions of this key lysine methyltransferase. Moreover, beyond the role of this enzyme in epigenetics and H3K36me3-dependent processes, we highlight the putative role of "non-epigenetic/H3K36me3" functions of SETD2 in cancer, particularly those involving the cytoskeleton.

## Introduction

The methyltransferase SET (*Su(var) 3-9) Enhancer of zest and Trithorax*) domain-containing 2 (SETD2) is involved in the epigenetic modification of histone H3. More specifically, SETD2 is the unique tri-methyltransferase specific for lysine 36 (H3K36me3). In yeast, it is also known to mono- and di-methylate H3K36. However, this activity was not found *in vivo* in mammals albeit such activity has been reported using *in vitro* conditions [1]. SETD2 was initially described to bind Huntingtin, and the complex between SETD2 and Huntingtin was then demonstrated to be essential for the translocation of SETD2 from the nucleus to the cytoplasm [2]. SETD2 is ubiquitous, non-redundant, exclusive of H3K36 in nucleosomes and its genetic invalidation is lethal at the embryonic stage and deleterious for the development of the vascular system [3]. SETD2 is involved in various aspects of the control of gene expression (activation and repression of transcription, or even repression of initiation of cryptic transcription [4]) as well as splicing, DNA repair and DNA methylation through its lysine methylation activity and through protein-protein interactions *e.g.* with DNA, histones and other proteins forming platforms at nucleosomes [5].

SETD2 is a Protein Lysine Methyltransferases (PKMTs) that catalyze histone and non-histone methylation at lysine residues. In humans, a super-family of approximately 50 PKMTs shares a 130 residues SET domain catalyzing lysine methylation. An important feature of the SET domain is the presence of distinct but closely related substrate and S-adenosyl-L-methionine (SAM) binding regions, which enable the transfer of the methyl group by a nucleophilic substitution (SN_2_) mechanism [6,7].

**Fig. 1:**
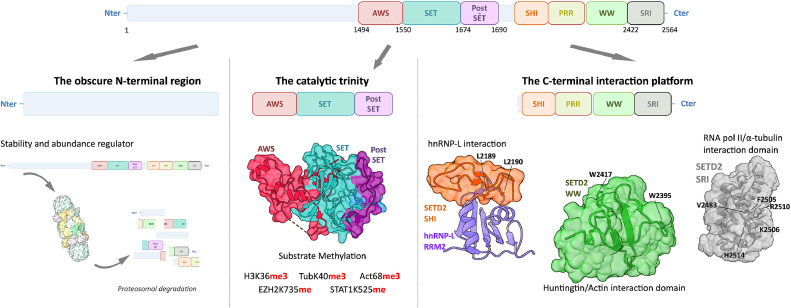
Structural and functional representation of SETD2 regions and domains. Schematic representation of the 3 functional regions of SETD2. The N-terminal region (left) responsible for regulating SETD2 stability, the catalytic region (middle) and the C-terminal region (right) acting as an interaction platform. *Left panel:* The N-terminal region of SETD2 has no clearly defined domain or structural organisation. It is known to regulate the degradation of SETD2 by the proteasome (figure done with PDB entry 1FNT) and the initiation of the phase separation process [10,30]. *Middle panel:* The catalytic region of SETD2, composed of the AWS, SET and post-SET domains, is responsible for substrate methylation by SETD2. Structural studies of this region have shown the importance of the SETD2 SET and SETD2 post-SET domains for substrate binding and catalysis, and the supporting role of SETD2 AWS to stabilise the SET catalytic domain (PDB 5JJY) [11]. *Right panel:* The C-terminal region of SETD2 contains 4 domains (SHI, PRR, WW and SRI). The SETD2 SHI domain mediates interaction with hnRNP-L through SETD2 L2189 and L2190 residues (PDB 7EVR) [41]. The SETD2 WW domain forms a globular structure (PDB 2MDC) and recognises Pro-rich regions that lead to interaction with huntingtin and actin, by SETD2 W2395 and W2417 residues [19]. The SETD2 SRI domain, composed of 3 helices (PDB 2A7O), links SETD2 to transcription regulation by interacting with RNA pol II [32], and to genome stability by interacting with α-tubulin *via* SETD2 V2483, F2505, K2506, R2510 and H2514 residues.

Surrounding the SET domain, two Cysteine-rich subdomains have been identified as essential for enzymatic activity (probably mostly by stabilising the SET domain structure) (Fig. 1). Interestingly, despite the importance of these subdomains, they are not strictly conserved within the SET PKMTs super-family [8]. At the C-terminal end of the SET domain of SETD2, a post-SET domain is present and is also required for optimal catalytic activity. The N-terminal side of the SET domain is flanked by the Associated With SET (AWS) domain which is needed for activity and has been shown to be involved in SETD2 proteasomal degradation. Interestingly the AWS domain is also able to interact with nucleosomal DNA and histone H3^9^.

The N-terminal domain of SETD2 is now recognized to be dispensable for enzymatic activity but to be involved in proteasome-associated degradation of the enzyme [10]. The presence of intrinsically disordered regions (IDRs) in this domain is proposed to participate in its stability but most interestingly to be involved in a process of aggregation initiating the formation of phase-separated liquid droplets. The C-terminal domain of SETD2 harbors 4 subdomains forming platforms for interactions with different molecules. The Set2 Rpb1 Interacting (SRI) domain interacts with RNA polymerase II (RNA pol II) and is implicated in the regulation of RNA transcription. This SRI domain is also involved in tubulin interaction. Further on the C-terminal the SETD2 hnRNP Interaction (SHI) domain is involved in splicing and transcription processes by recruiting hnRNP-L to the site of transcription by RNA pol-II. Finally, the SETD2 Tryptophans (WW) and the autoinhibitory Proline-rich region (PRR) domains are involved in interactions with various proteins from which Huntingtin was the first to be characterized.

Strikingly SETD2 has been further described as interacting with proteins outside the nucleus, particularly with the cytoskeletal proteins tubulin and actin [11] but also with cell-signaling elements (such as STAT1, Talin, VAV1), epigenetic enzymes (EZH2) and transcription factors (p53) [12]. SETD2 is now well depicted to interact with and to tri-methylate α-tubulin specifically on lysine 40 (α-TubK40me3), thereby modifying microtubule dynamics. Interestingly, lysine 40 can also be acetylated to form α-tubK40ac which is known to modulate microtubule properties. These two mutually exclusive post-translational modifications are negatively cross regulated. Altogether these interrelated post-translational modifications (PTM) of various protein substrates may constitute a regulation network of both genome expression and cell activation.

**Fig. 2:**
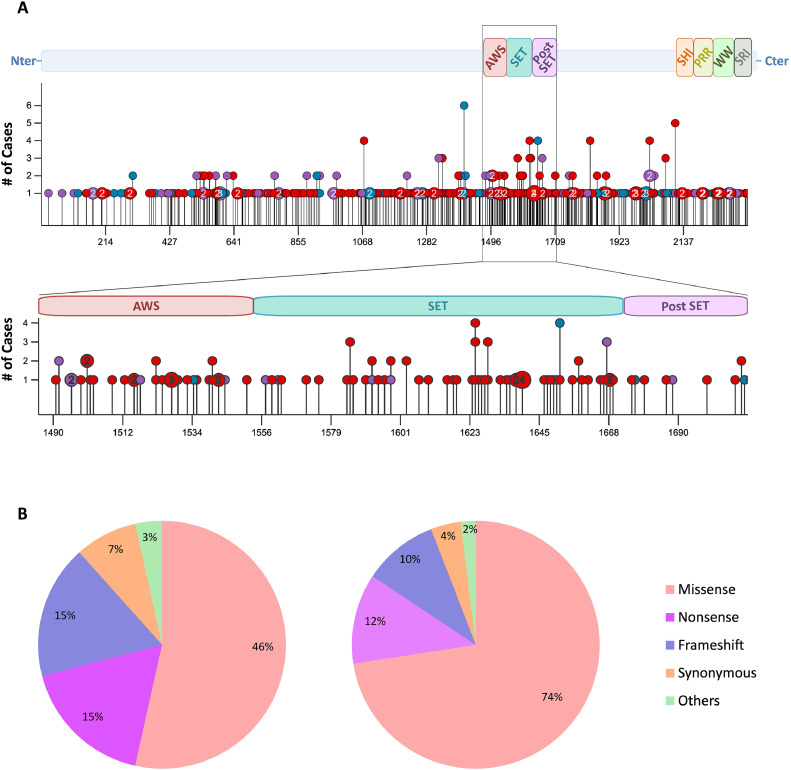
SETD2, a cancer driver gene. A: Identified cancer-associated mutations, as documented by the The Cancer Genome Atlas (TCGA) database, in the complete SETD2 enzyme and catalytic region (AWS, SET, post-SET). Missense mutations are shown in red, nonsense mutations in purple and frameshift mutations in blue. B: Overview of the distribution of mutations in SETD2 enzyme (left) and catalytic region (right), as described in the COSMIC (Catalogue Of Somatic Mutations In Cancer) database.

SETD2 is associated with various cancers and is considered as one of the most frequently mutated genes in tumors [[13], [14], [15]]. Indeed, various mutations along the entire sequence of the protein have been reported. Most of these alterations are likely loss-of-function mutations that could affect the methyltransferase activity of the enzyme as well as its involvement in molecular assemblies (Fig. 2). As stated above, numerous tumors are associated with *SETD2* genetic alterations, including clear-cell renal carcinomas which show the highest mutation frequency among *SETD2*-related cancers, but also leukemias and lymphomas, non-small-cell lung tumors, gastric tumors and gliomas. Although most mutations affecting *SETD2* are monoallelic and may lead to haploinsufficiency, biallelic genetic defects in *SETD2* and genetic translocations are found in different cancers [[16], [17], [18]].

It thus appears that SETD2 may play a key role in the regulation of the histone and cytoskeleton codes through its methylation activity. Its involvement in controlling these post-translational modifications makes this enzyme a regulator of the functionality of cytoplasmic microtubules and nuclear chromatin, and thus a key element of the mechanisms of oncogenesis [11].

**Fig. 3:**
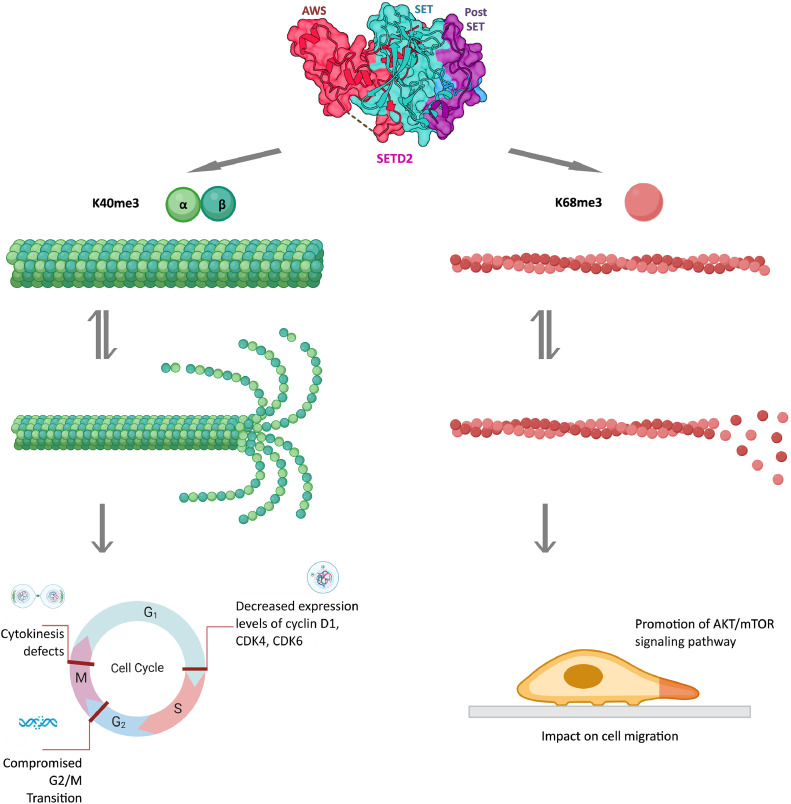
Schematic representation of the methylation of microtubules (green structure) and actin (red structure) by SETD2. The catalytic region of SETD2, composed of the AWS, SET and post-SET domains is shown. The loss of methylation of K40 of the alpha subunit of microtubules is often associated with catastrophic microtubule defects and several defects in the cell cycle. Actin trimethylation (ActK68me3) by SETD2 has been associated with actin dynamic equilibrium between polymerization and depolymerization and with cell migration.

Interestingly, in addition to microtubule, SETD2 has recently been shown to regulate the actin cytoskeleton, through its lysine 68 methylation activity (ActK68me3) of Globular actin (G-actin) promoting its polymerization into Fibrillar actin (F-actin) [19]. SETD2 therefore appears to be a critical control point at the convergence of epigenetic process, cell cycle and cell motility (Fig. 3). Beyond its H3K36 methyltransferase activity, it is thus essential to explore the interplay of SETD2 and its non-histone protein substrates on gene expression, cell division or cell migration, particularly in carcinogenesis. To this end, it is essential to gain a better understanding of SETD2 structural domains and their functions. The description of SETD2 domains and residues involved in known functions of the protein are detailled herein through analysis of tumor-associated mutations.

### Structural and functional analysis of SETD2 domains

#### Substrate recognition by the catalytic “trinity” of SETD2

##### The SET catalytic domain

To characterize the interaction between SETD2 and its histone substrate (H3), structural analyses have been carried out in recent years, using crystallography [20,21] and electron microscopy [9]. To document this interaction, residue K36 was replaced by a methionine (H3K36M), as H3K36M histones are known to act as an “oncogenic inhibitor” of SETD2 activity and H3K36M peptides display increased binding to the active site of the enzyme [22]. According to these studies, SETD2 substrate is positioned in the active site of the enzyme through the formation of a pseudo-β-sheet, resulting from interactions with the L_α6β5_ and L_IN_ loops (auto-inhibition loop).

**Fig. 4:**
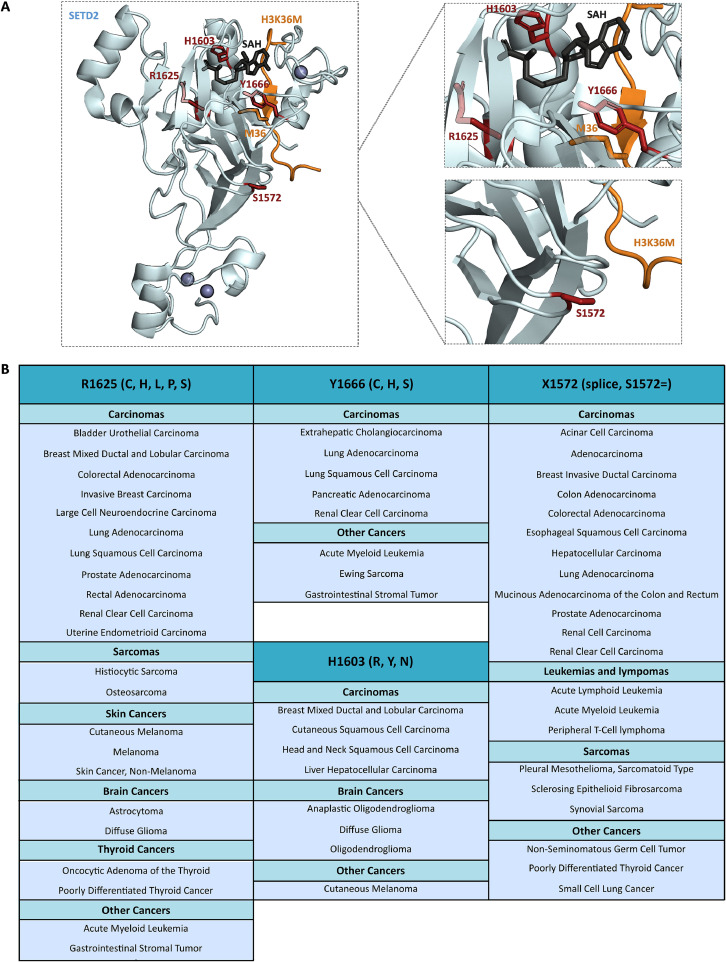
SETD2 mutation hotspots, mapping their position in the structure and the cancers in which they are involved. A: SETD2 catalytic domain (AWS – SET – post-SET) crystal structure (PDB 5JJY) [20] in blue cartoon. SETD2 mutation hotspots S1572, H1603, R1625, Y1666 are highlighted in red sticks. H3K36M peptide substrate is represented in orange cartoon and M36 residue is in sticks. SAH cofactor is represented in black sticks. *Left panel:* Overall structure. *Right panel:* Closed view of SETD2 mutation hotspots. B: Classification of cancers with *SETD2* gene mutation hotspots as documented by cBioPortal for cancer genomics database.

In the different structures of SETD2 catalytic domain in complex with H3K36 peptide or nucleosome (mimicking the target lysine 36), the interaction of SETD2 with residue M36 is stabilized by four residues that form a hydrophobic cage around the target lysine (Y1579, M1607, F1664, Y1666). Among these residues, Y1666 is described as a mutation hotspots suggesting a major role of this residue although no studies have been conducted yet (Fig. 4). In addition, the other residues flanking H3K36M reinforce this recognition by hydrogen bonds and hydrophobic interactions. Histone H3 residues A29-V35, being small and uncharged, fit into a narrow electropositive pocket (formed by SETD2 residues F1589, Y1604, F1606, F1668), whereas histone H3 H39-Y41 residues form hydrogen bonds with residues E1636 and T1637 of SETD2, thanks to the *trans* configuration of residue H3P38. The H3P38 *cis* configuration, but also H3P38V mutations, are known to impair the interaction between SETD2 and H3 suggesting the importance of P38 for substrate binding [21].

A particular feature of the recognition of residue H3K36 by SETD2 is the presence of two glycine residues close to the H3K36 (G33-G34). The structure of SETD2 shows that these two glycine residues, perfectly fit into a narrow tunnel formed by the aromatic rings of residues Y1604, F1668, Y1671 and the main chains of residues Q1669-G1672. Any other amino acid in this position is incompatible with this tunnel and does not allow the enzyme to bind to its substrate. Importantly, H3G34 is mutated in 50 % of pediatric gliomas and H3G34V/R/D cancer driver mutations are proven to be incompatible with SETD2 substrate binding [21,23]. Mutation of H3G34 into a residue with a bigger side chain blocks the SETD2 H3K36 interaction by steric hindrance and abolishes H3K36 trimethylation by *cis*-inhibition. As a result, the recruitment of mismatch repair machinery (MMR), by MSH6 recognition of H3K36me3, is abolished and cells expressing H3G34V/R/D mutations present a similar phenotype with deficient MMR pathway and a high mutation frequency [23].

Although SETD2 has been considered as a specific H3K36 methyltransferase, non-histone substrates of SETD2 have been discovered over the last few years (some of which will be presented below). Using peptide array, Schumacher *et al.* [24] identified a new artificial peptidic “super substrate” of SETD2 (ssH3K36). They demonstrated that SETD2 strongly prefers amino acids different from those that surrounds H3K36. This work allowed to determine a new specificity profile for SETD2 substrates, corresponding to the motif (R, I, L, Y, F) - G - (F, G > A, Y) - (I, V, L, F, Y) - **K**- R (not E, G, P) - (A, I, P > G, L, S, T, V), where **K** is the target substrate lysine. More recently, we provided structural and enzymatic evidence that SETD2 trimethylates tyrosine kinase ACK1 *in vitro* [25]*.* This methylation (ACK1 K524) occurs in a motif that fits to the model proposed by Schumacher *et al.* [20].

The binding of this "super substrate" to SETD2 (ssH3K36) has been further established using molecular dynamics simulations and FRET assays [26]. The ssH3K36 peptide substrate association seems to be enhanced by a hairpin conformation. Once the SETD2-substrate complex is formed, additional contacts between the peptide substrate and SETD2 are established towards C- and N-termini of the peptide in a zipper-like process. This leads to an unfolding of the hairpin and adoption of an extended peptide conformation, which is equally important for the methylation of SETD2 substrate.

Furthermore, Cryo-EM analysis of SETD2 bound to nucleosomes (WT or H3.3K36M) suggests that the interaction of SETD2 with the WT nucleosome is highly dynamic and flexible [9]. In order to establish a SETD2-nucleosome interaction, DNA is unwrapped from the core histones. This unwrapping is crucial for the interaction between SETD2 and the nucleosome partners, as it exposes the SETD2 binding site, similar to the Pol II passage during transcription elongation. Interestingly, these two processes, SETD2 binding (allowing deposition of H3K36me3) and transcription elongation, are well known to be linked. This process is inhibited by the presence of histone H1, which reinforces the compact conformation of the nucleosome and prevents DNA unwinding. Indeed, the distributions of H1 and H3K36me3 appear to be mutually exclusive in chromatin.

##### The “catalysis supporters” AWS and post-SET zinc finger subdomains

The AWS subdomain, flanking the N-terminus of the SET domain, is characteristic of the SET family H3K36 methyltransferases (such as SETD2, NSD1, NSD2 or NSD3), suggesting that it is probably specific for methylation of residue H3K36. It comprises around 50 residues, including seven cysteine residues, which coordinate two zinc atoms (Zn^2+^). These elements form an unusual Zn2C7 zinc finger structure. The AWS domain was discovered during computational tests aimed at identifying new protein domains [27]. Its precise function remains poorly documented, despite its importance for enzymatic activity. However, it is assumed that it stabilizes the structure of the SET domain by interacting with residues distant from the catalytic site. The resolution of the cryo-EM structure of the SETD2 catalytic domain linked to nucleosomes by Liu *et al*. showed that residues of the AWS domain interact with residues Y41, R49 and R52 of H3, which reinforces the idea of the importance of this domain in enzymatic catalysis [9].

The post-SET subdomain is located at the C-terminal end of the SET domain and takes the form of a flexible loop. It is also composed of a C4-type zinc finger, formed by four cysteine residues, three of which are in the post-SET domain and the fourth in the C-terminal part of the SET domain (C1631). It is interesting to note that the positioning of H1629 (SET domain) suggests the potential presence of a fifth coordinator residue of this zinc finger. The post-SET domain loop (also known as the autoinhibitory loop or L_IN_) can adopt several conformations that allow the enzyme to be self-regulated, notably through the position of residue R1670, which can switch to the substrate-binding site and occupy the target lysine-binding position in a close conformation [20,21,28]. This auto-inhibition process has also been identified in NSD1 and ASH1L, two other SET2 family members, suggesting that it could be an autoregulatory mechanism of H3K36 methyltransferases. In addition, residues of the post-SET domain (such as F1668, Q1669, R1670, Y1671 and G1672) are also involved in the formation of substrate and SAM cofactor binding pockets [20,21,28]. Interestingly, the post-SET domain is unstructured in the apo form of the enzyme (without H3 binding). Upon substrate binding, this domain folds (with the coordination of a Zn^2+^ atom and the formation of a loop around the substrate) in order to stabilise enzyme/substrate interaction.

#### The “obscure” N-terminal region of SETD2

The N-terminus of SETD2 (residues 1-1443) has long been the 'dark side' of the enzyme, as no specific role has been associated with it. Moreover, this domain has been characterized as not being required for H3K36 methylation activity [29]. In 2020, Bhattacharya and Workman demonstrated that this region is a regulator of SETD2 half-life and stability through aggregation and proteasomal degradation [30]. Removal of SETD2 N-terminus leads to stabilization of the enzyme and increased deposition of the H3K36me3 in an RNA-pol II independent manner, suggesting that regulation of the half-life of SETD2 is important for its function. Stabilization of SETD2 also leads to increased cell proliferation, although it is considered a tumour suppressor. However, no effect of SETD2 accumulation on its non-histone targets has been demonstrated yet.

Because of the importance of regulating SETD2 expression, there are multiple controls on its abundance that prevent spurious deposition of H3K36me3. It is now known that SETD2 is composed of IDRs. In total, SETD2 has 17 IDRs, 11 of which are located in the N-terminal part and regulate the enzyme expression level. These motifs/regions do not contain a specific domain or sequence similarity with other known proteins and are constituted of charged amino acids with low hydrophobicity [10]. The lack of a defined structure makes these regions more susceptible to proteolysis, particularly by the proteasome.

IDRs are known to form protein phase separation as they act as flexible platforms for protein-protein interactions. Once nucleation has started, other IDRs interact, leading to the formation of phase-separated liquid droplets. In the case of SETD2, the IDRs present in the C-terminal part of the enzyme (which also contains the catalytic domain), are those that promote phase separation when the N-terminal region is absent. However, the mechanism of this initiation and the differences between the SETD2 N- and C-terminal IDRs remain unclear. Therefore, the N-terminal region of SETD2 controls the abundance of the enzyme by regulating its degradation by the proteasome and preventing the formation of phase separation droplets [10].

Until the very recent work of *Khan et al.* no other role had been attributed to the N-terminal region of SETD2 [31]. *Khan et al.* demonstrated the presence of a phenotype due to the loss of SETD2, which is not linked to the loss of methylation activity of the enzyme, as they showed that the N-terminus of SETD2 is important for the association of CDK1 with lamins. In fact, the absence of SETD2 or its N-terminal region leads to a reduction in lamin phosphorylation and an alteration in nuclear lamin dynamics. This suggests a destabilization of the integrity of the nuclear lamina and genome instability, which could enable SETD2 to play its role as a tumor suppressor by controlling the disassembly of the nuclear envelope during the cell cycle [27].

#### The SETD2 C-terminal interaction platform

In contrast to the N-terminal region, the C-terminal region of SETD2 has been better elucidated. In addition to the catalytic core (AWS - SET - post-SET), the C-terminal region of SETD2 contains four additional domains and acts as a platform for interaction between SETD2 and molecular partners.

##### The Set2 Rpb1 interacting (SRI) domain

The SRI domain, originally discovered near the C-terminus of SETD2 [32] and in the helicase RECQL5 [33], is involved in the interaction of these enzymes with the RNA polymerase II (RNA pol II) and in the regulation of the RNA transcription process. This small domain, spanning residues 2422-2564 in SETD2, interacts with the largest subunit of RNA pol II in eukaryotes, the CTD (C-Terminal repeat Domain) which is composed of multiple repeats of the sequence Y_1_S_2_P_3_T_4_S_5_P_6_S_7_. At the beginning of the transcription process, the CTD domain is not phosphorylated and, during the elongation process, it is phosphorylated at serine 2 and 5. The hyperphosphorylated form of CTD is known to be recognized specifically by the SRI domain [32].

SETD2 is recruited by RNA pol II *via* its SRI domain and enables transcription to progress, in particular through the formation of the H3K36me3 mark. Resolution of the structure of the SETD2 SRI domain showed that it is organized into 3 α-helices, allowing it to be differentiated from the other CTD interaction domains that have been described to date [34,35]. The interaction of SETD2 SRI with the CTD domain of RNA pol II involves C-terminus SETD2 α1 and α2 helices and more specifically residues V2483, F2505, K2506, R2510, H2514. The positive chains of K2505, R2510 and H2514 interact with phosphorylated serine 2 and 5 of RNA pol II, while the hydrophobic residues V2483 and F2505 form a hydrophobic patch in a groove formed by the α1 and α2 helices of the CTD domain [34,35].

**Fig. 5:**
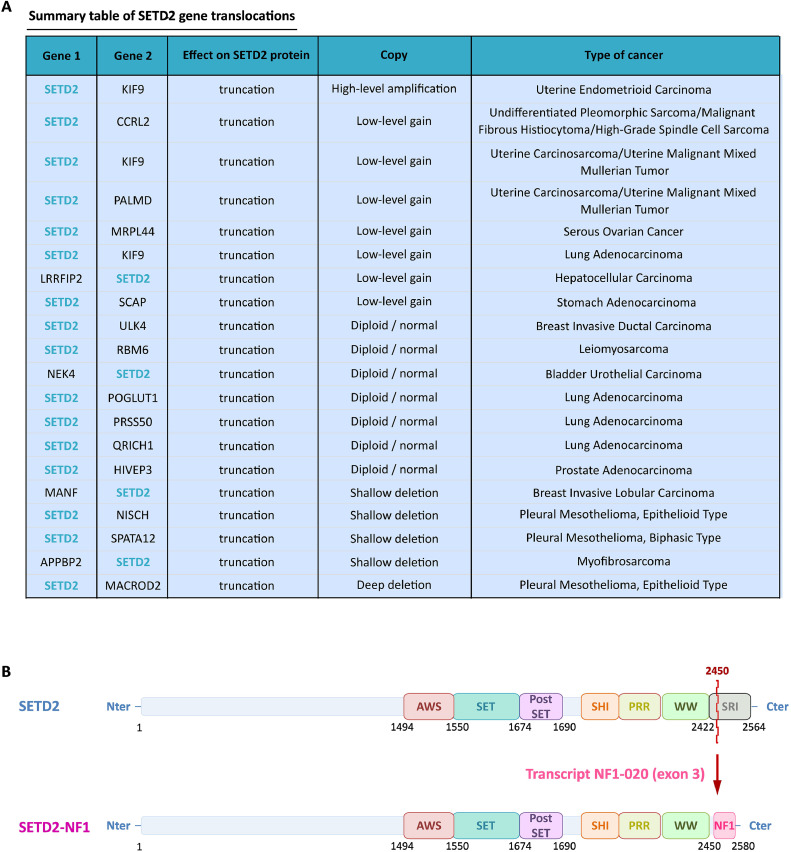
SETD2 gene translocations. A: Translocations of the *SETD2* gene and types of cancer in which they are involved. Non-exhaustive table compiled by cBioPortal for the Cancer Genomics Database. B: A case of *SETD2* translocation described in a paediatric cell spider tumour with chromosomal translocation t(3;18)(p21;q12) and loss of the SETD2 SRI domain. SETD2 (1-2450) fuses with exon 3 of the NF1-020 transcript to form the SETD2-NF1 fusion protein[37].

The interaction between SETD2 and RNA pol II does not appear to affect SETD2 enzymatic catalysis, since overexpression of the R2510H oncogenic mutant has no impact on SETD2 methylation activity nor the stability of the enzyme [29]. Nevertheless, deletion of the SETD2 SRI domain results in an inability to recruit SETD2 to its target gene locus through binding to the CTD domain of RNA polymerase II. This hypothesis was confirmed by the study of the truncated mutation p.Gln2325*, which results in a global loss of H3K36me3 level [36]. In a same way, the *SETD2-Nf1* fusion gene (Fig. 5), identified in a paediatric spindle cell tumour with the chromosomal translocation t(3;17)(p21;q12), results in deletion of the SETD2 SRI domain and a decrease in H3K36me3 level [37].

Recent studies have shed light on the molecular determinants of α-tubulin recognition by SETD2. It has been shown that the SRI domain of the enzyme recognizes α-tubulin. The R2510H mutation in SETD2 abolishes tubulin binding and methylation, suggesting a role for the SRI domain in α-tubulin binding, although the SETD2 catalytic domain was initially described as sufficient for tubulin binding [11]. This SETD2 SRI – α-tubulin interaction relies on different residues than those involved in the SETD2 SRI – RNA pol II, since F2505A binds tubulin but not RNA pol II, and R2510H binds RNA pol II but not tubulin. However, as SETD2 SRI binds to the negatively charged CTD domain of RNA pol II, recognition of α-tubulin is also achieved by the charged residues of the SRI domain interacting with the negatively charged C-terminal region of tubulin [38].

##### The SETD2 hnRNP Interaction (SHI) domain

It has been shown that the splicing and transcription processes are linked [39]. As SETD2 is linked to these two cellular processes, it is considered to be an ideal candidate for facilitating the splicing process, through its ability to recruit heterogeneous nuclear ribonucleoproteins such as hnRNP-L to the site of transcription by RNA pol-II [40]. In addition, SETD2 and hnRNP-L partially regulate the transcription and splicing processes of the same genes, which show enrichment of the SETD2 signature mark, H3K36me3 [41]. SETD2 has a hnRNP-binding domain, called SHI (SETD2 hnRNP Interaction) which is composed of residues 2164-2263. The SETD2 SHI domain is a small, disordered domain that most often interacts with hnRNP-L *via* the RNA recognition motif 2 (RRM2) domain of hNRP-L, independently of RNA pol II. Loss of the SHI domain also results in decreased trimethylation of H3K36 [40,41].

Bhattacharya *et al.* solved the crystallographic structure of RRM2 of hnRNP-L in complex with SETD2 SHI domain (residues 2167-2192; PDB: 7EVR) and described the molecular determinants of the SETD2 SHI – hnRNP-L interaction [42]. Once the two partners interact, SETD2 SHI adopts a U-shaped conformation that contacts the dorsal helical face of the RRM, with SETD2 residues ^2183^NAGKVLLPTP^2192^ forming direct contacts with RRM2. SETD2 L2188 and L2189 play an important role in this interaction, as they are embedded in a hydrophobic cage and apolar surface of RRM2 respectively. Residues L2189-P2190-T2191-P2192 of the SHI domain also participate in this interaction by wrapping around RRM2 Y204 and Y257. The crystal structure also showed the possible formation of a ternary complex between SETD2, hnRNP-L and RNA, given that the binding sites of hnRNP-L with the other two partners do not overlap.

##### The SETD2 tryptophane (WW) and the autoinhibitory proline rich region (PRR) domains

The WW domain was involved in the first documentation of the interaction of SETD2 with Huntingtin, the enzyme being originally described as Huntingtin-interacting protein B (HYPB) [2]. WW domains are a small, conserved protein motif involved in protein-protein interactions. It is composed of 35 to 40 residues and named after the two strictly conserved Tryptophan (Trp) residues spaced 20 to 22 amino acids apart (W2395 and W2417 for SETD2). After folding, it adopts a stable globular structure, made up of three antiparallel β strands surrounding the hydrophobic core [43]. This structure recognizes proline-rich regions (PRR), in particular PPxY or PPLP [44] motifs. The SETD2 WW – Huntingtin interaction has also recently been described as the mediator of the link of SETD2 with cytoskeletal actin, resulting in actin methylation at lysine 68 (ActK68me3). Disruption of this axis leads to loss of ActK68me3 and subsequent defects in actin depolymerization and impaired cell migration [19].

It is interesting to note that SETD2 has a PRR domain, located upstream of the WW domain. This domain contributes a form of allosteric self-regulation of SETD2 interaction with its partners, in particular the Huntingtin protein (HTT). Notably, the WW and PRR domains of SETD2 can interact within the same molecule, inducing a closed conformation, six times more abundant than the open conformation, which inhibits the interaction of the SETD2 WW domain with other PRRs[45]. The central WW domain contacts the PRR domain through the β3 strand and the loop between the β1 and β2 strands. In addition, the SETD2 WW domain participates in the redistribution of the SETD2 from the nucleus to the cytoplasm (by the HTT interaction), thus this relocation can be regulated by the SETD2 PRR domain [45].

SETD2 belongs to the family of factors capable of modifying and remodeling chromatin and the cytoskeleton. Hence, SETD2, *via* its binding to microtubules and actin, is capable of regulating both their structure and their function. This dual function, known as “chromatocytoskeletal activity”, integrates nuclear and cytoplasmic functions and regulates numerous cellular processes [46]. In the following paragraphs we describe the role of SETD2 in cell proliferation and migration, and the pathological consequences of this involvement, particularly in cancer.

### Cell cycle regulation and proliferation

SETD2 trimethylates both histone at H3K36 and α-tubulin at K40 and its protein level is known to be minimal in G1 and maximal in G2/M [47]. Interestingly, during the M phase, tubulin and histone methylation promote cytokinesis and the separation of one cell into two daughter cells. Moreover, deficiency of SETD2 causes cytokinesis defects and cell cycle delay [48,49] (Fig. 3). Loss of SETD2 also leads to loss of microtubule methylation that is often associated with catastrophic microtubule defects [11]. Notably, methylation seems to be of major importance for activation of the NoCut checkpoint which depends on Aurora B at the spindle midzone and to complete cytokinesis [50]. Hence SETD2 could have a protective role in maintaining genomic stability, double-strand-break resection and homologous recombination repair [11]. These results suggest regulations and cross-talks between the cytoskeleton and epigenome and encourage further studies to better understand these phenomena in both normal and pathological conditions such as cancer.

The proper cell cycle progression allows the dynamic coordination of an associated organelle, the primary cilium. This process mainly relies on several cyclin-dependent kinase (CDK) as CDK4 and CDK6 and their corresponding cyclins. It has been shown that SETD2 deficiency caused S-phase arrest and compromised G2/M-phase transition in myoblasts but also G1-phase arrest (Fig. 3). The S-phase transition is also jeopardized in myoblasts C2C12 cell line [51]. Moreover, decreased expression levels of major regulators of cell cycle G1/S checkpoints, cyclin D1, CDK4, CDK6, and cyclin E2 were also detected under these conditions as CDK inhibitor p21 is significantly upregulated in *SETD2*^-/-^ in C2C12 cell line [51]. However, whether SETD2 regulates primary cilia biogenesis via the methylation of α-tubulin at K40 needs to be further documented.

More generally, SETD2, through its action on the cell cycle, is involved in proliferation and differentiation. Indeed, in myoblasts, staining for Ki67, a marker for cell proliferation, shows less proliferating cells in *SETD2*^−/−^ C2C12 cells [51]. These cells also revealed a deficiency in myoblast differentiation with a decrease in myotube formation in consistency with a lower expression level of MyoG and promoter activity [51]*.*

This regulation has also been observed in adult hematopoietic stem cells notably in *SETD2* conditional knockout allele mice [52]. Indeed, constitutive knockout of *SETD2* resulted in embryonic lethality at E10.5–E11.5 due to defects in the vascular architecture [18]*.* These mice have 30 % fewer nucleated bone marrow cells, enlarged spleen and obviously shrunken thymus. *SETD2* deficient mice also show a profound reduction of myeloid, lymphoid, and megakaryocytic progenitors, but significantly increased erythroid progenitors. More generally, the results demonstrate that SETD2 KO mice have intrinsic defects in bone marrow reconstitution with reduced hematopoietic stem cell self-renewal and quiescence but on the opposite an increased proliferation and commitment to progenitors [53]. This phenomenon has been hypothesized to be due to the upregulation of Gata1, Gata3, Klf1, and Myc proteins through enhanced pol II elongation [52] but also to the activation of activated E2F gene regulatory network and the reduced expression of the ribonucleotide reductase subunit Rrm2b [53]*.*

As discussed below, SETD2 has been associated with multiple cancers. In the case of lung adenocarcinoma, it has been shown by Zhou *et al.* that the tumorigenic process can be related to the regulation of the cell cycle by SETD2 [54]. Indeed, it has been shown that elevated chemokine CXCL1 levels promote cell cycle progression and this study has suggested that SETD2 activates CXCL1 through the methylation of its promoter hence affecting cell proliferation.

The importance of actin methylation also appears to be crucial in regulating many cellular processes. Indeed, it has been shown in 786-O kidney adenocarcinoma cell line that trimethylation favors actin polymerization and branched actin. This would facilitate the interaction with WHAMM, a nucleation-promoting factor that is known to interact with both actin and microtubules thus regulating not only endocytosis but also autophagy. Therefore, in cells lacking SETD2, a defect in autophagy has been observed and the possibility that SETD2-mediated regulation of actin polymerization could regulate endocytosis should be addressed [55].

### Cell migration

Actin trimethylation (ActK68me3) by SETD2 has been associated with actin dynamic equilibrium between polymerization and depolymerization and with cell migration (Fig. 3). This trimethylation depends on an interaction with Huntingtin protein (HTT) via the WW domain of SETD2 and the actin-binding adapter HIP1R. Moreover, it has been shown that disruption of the SETD2-HTT-HIP1R axis inhibits actin methylation, causing defects in actin polymerization. These effects on actin notably impact cell migration [19]. This defect has also been reported by Li *et al*. in keratinocytes. Indeed, they demonstrate that SETD2 deficiency is responsible for an accelerated re-epithelialization during cutaneous wound healing through the promotion of proliferation but also migration through the activation of AKT/mTOR signaling pathway [56] (Fig. 3).

These results have been reinforced by the work of Yang et al. who have combined data mining of public patient data sets and human lung adenocarcinoma tissue arrays and lung cancer cell lines [57]. Their findings have also underlined the suppressive role of SETD2 in cell proliferation and migration but furthermore its suppressive role in invasion and epithelial-mesenchymal transition (EMT). These last processes would be caused in lung adenocarcinoma by the negative regulation of IL-8 transcription in a STAT1-dependent manner relying on SETD2 trimethylation of H3K36 [57]. However, in renal proximal tubule epithelial cells (RPTEC) CRISPR KO for *SETD2*, it has been shown that the promotion of invasion, migration, EMT and stemness would be triggered in part by cytokines and growth factors but also by transcription factors including SOX2, POU2F2 (OCT2), and PRRX1 [58]**.**

### Cancer

Dysregulated histone methylation is often associated with the early process of tumor development [59,60]. Because of its role in proliferation, migration, and invasion, SETD2 is routinely associated with cancer. Indeed, it is frequently mutated in numerous cancer types, for example, in up to 16 % of clear cell renal cell carcinomas (ccRCC) and can be as high as 30 % in metastatic ccRCC [59,60]. SETD2 is also frequently mutated or under expressed in solid tumors such as breast tumors, pediatric high-grade gliomas, lung carcinomas, gastric/pancreatic ductal adenocarcinomas, and colorectal carcinomas but also leukemia and lymphoma. Many different consequences have been observed such as defects in transcriptional elongation, deficiency linked to p53 and alterations in different signaling pathways[61].

Cancer gene databases have identified four mutation hotspots in the SETD2 gene. Interestingly, these four residues (X1572, H1603, R1625 and Y1666) are located in the SET catalytic domain. As shown in Fig. 4, they are in close proximity to the substrate, cofactor and/or zinc finger, suggesting an effect on enzymatic activity, although no studies have yet been carried out with the exception of the R1625C mutation [29].

Furthermore, *SETD2* gene translocations have been described in several types of cancers [62,63]. Hence, in a pediatric spindle cell tumor SETD2 has been found to be fusionned to NF1 with the chromosomal translocation t(3;17)(p21;q12) and loss of SETD2 SRI domain. The fusion transcript produces a less active form than full-length SETD2, as the lack of interaction with RNA pol II results in impaired recruitment of SETD2 to the gene locus [37] (Fig. 5).

#### Leukemia and lymphoma

SETD2 has early been identified in CD34+ cells [64,65]. Modifications of *SETD2* are the most common genetic changes detected in monomorphic epitheliotropic intestinal T-cell lymphoma and hepatosplenic T-cell lymphoma. Defects in *SETD2* have been also detected in many lymphoid malignancies such as precursor B-cell lymphoblastic leukemia, early-T-precursor lymphoblastic leukemia, diffuse large B-cell lymphoma, chronic lymphoblastic leukemia, small lymphocytic lymphoma [66] and even in mast cell leukemia [67]. Moreover, our recent data indicate that benzoquinone, a major hematotoxic metabolite of benzene, could contribute to benzene-dependent leukemogenesis by perturbing the functions of SETD2[68].

*SETD2* mutations have been reported in 6 % of acute leukemia with 22 % enriched in MLL (Mixed Lineage Leukemia)-rearranged leukemia [18,52]. MLL-fusion proteins are found in acute lymphoblastic leukemia (ALL) and acute myeloid leukemia (AML) and are often associated with poor prognosis, notably in pediatric tumors [69]. As seen above, one reason for SETD2 being involved in MLL leukemia may be because *SETD2* deficiency induces DNA replication stress in Hematopoietic Stem Cells (HSCs) which results in proliferation and cell cycle abnormalities, genomic instability, hence leading to the accumulation of secondary mutation(s) that synergistically contributes to tumorigenesis [36,53]. For example, recurrent deletions of the *SETD2* locus were found in 3 % to 4 % of chronic lymphocytic leukemia (CLL) patients and these deletions are associated with loss of TP53, genomic complexity and chromothripsis. This is in line with the role of SETD2 as a tumor suppressor since early loss-of-function events in CLL pathobiology are currently observed and linked to aggressive disease [70].

*SETD2* has also been shown to be the most mutated gene in Enteropathy-associated T cell lymphoma (EATL) (22 of 69 cases), Moffitt *et al*. suggesting that the mutations observed favor, here again, a loss-of-function of *SETD2* [71]. Interestingly, *SETD2* deficiency is accompanied by an expansion of γδ T cells, suggesting a novel role for SETD2 in T cell development, in addition to its role in oncogenesis [71]*.* These results can be put into perspective with those obtained for EZH2 (enhancer of zeste homolog 2), a H3K27 methytransferase and substrate of SETD2, recently implicated in the regulation of the early stages of T-cell maturation through the control of the polarization of the Microtubule Organization Center (MTOC) [72].

Of note, studies from Skucha *et al*. underline that the role of SETD2 in leukemia could be more complex [69]. Indeed, heterozygous *SETD2* loss in leukemia cells expressing a MLL (Mixed Lineage Leukemia)-AF9 fusion protein was found to accelerates leukemogenesis driven by the MLL fused gene. In contrast, complete loss (homozygous) delayed leukemogenesis [69]. These results thus show that homo-versus heterozygous *SETD2* loss have not the same impact on leukemogenesis and suggest that SETD2 may function as an haplo-insufficient tumor suppressor and that homozygous loss of SETD2 hamper cancer progression. However, these opposite effects of SETD2 are likely context-specific [69].

#### Solid Tumors

##### SETD2 in renal cancer

Among the different types of renal cancer, ccRCC is the major type with a high incidence rate and poor prognosis. The observed mutations of *SETD2* were predominantly nonsense or missense mutations, frame shift and fusion, all leading to loss-of-function. In ccRCC several mutations of tumor suppressor genes and chromatin regulators have been identified near von Hippel-Lindau (VHL), including Polybromo1 (PBRM1), BRCA1 associated protein-1 (BAP1), and SETD2 [73]. Furthermore, *SETD2* deficiency is associated with ccRCC recurrence and poor prognosis. Hence, *SETD2* loss-of-function mutations were identified in 10 %∼20 % of primary ccRCC tumors, increasing to 30 %∼60 % of metastatic ccRCC tumors, H3K36me3 being reduced in distant metastases [74]. However, it is noteworthy that *SETD2* mutations seem to be acquired and selected from pre-existing VHL- and/or PBRM1-mutated cells [75]. SETD2 has been associated with multiple defects in ccRCC including cryptic transcription [4], RNA splicing [76], DNA repair [77] or even autophagy [78]. All these mutations and molecular processes have recently been reviewed in an exhaustive way by Yu *et al* [79].

The most mutated catalytic residue R1625 is described as a hotspot mutation in the COSMIC and CBioPortal databases. Hackeer et al. deeply studied the R1625C missense mutation, located in the catalytic domain of SETD2, near the SAM cofactor binding site (Fig. 4). This mutation leads to an inactive enzyme, due to altered substrate binding, as demonstrated by in silico and peptide pull-down assays. In addition, the mutated protein is less stable than the WT form in SETD2 KO transfected cells. Accordingly, human cells expressing the SETD2 R1625C mutation show a decrease in the H3K36me3 epigenetic mark and defects in DNA repair [29].

##### SETD2 in prostate cancer

*SETD2* mutations have been identified in prostate cancer and it significantly clusters in prostate cancer samples over-expressing androgen receptors. Notably, with NSD1, and ASH1L, two other H3K36 methyltransferases, SETD2 were demonstrated as a critical gene in the development of castration-resistant prostate cancer (CRPC), similar to MLL complex family members as described above for leukemia [80].

Several studies have demonstrated that DNA methylation plays a significant role in metastatic prostate cancers (PCa). For instance, it has been shown that EZH2, which tri-methylates histone H3K27 and induces gene silencing, is associated with poor survival [81]. In line with these results, Yuan *et al*. have demonstrated that the tumor-suppressive function of SETD2 in PCa is largely dependent on EZH2 modification. Hence, they showed that SETD2 directly methylates EZH2 at K735 and promotes its degradation and identified one recurrent mutation on SETD2 (R1523) in AWS domain abrogating the interaction but not the methylation of EZH2. Furthermore, it seems that AMPK signaling regulates SETD2 expression, suggesting that the SETD2-EZH2 axis translates metabolic pathways with consequences onto the chromatin state [82].

##### SETD2 in gastrointestinal cancer

Gastrointestinal cancer (GI) covers all cancers of the digestive tract, including gastric, pancreas and colon cancer. Generally speaking, in gastrointestinal stromal tumors (GIST) functional consequences of SETD2 mutations were investigated in primary tissues and cell lines and *SETD2* has been described as a novel GIST tumor suppressor gene associated with disease progression [83].

Hence, *SETD2* as *SETDB1* and *SETDB2* genes, two other H3K36 methyltransferases, are altered in gastric cancer (GC) [84]. Notably, there is a loss of about 80 % of mRNA expression in tumor tissue compared with adjacent normal tissue [60]. This lower expression is correlated with a poor prognosis and SETD2 overexpression in GC cell lines significantly inhibited cell proliferation, migration, and invasion hence reinforcing the hypothesized role of tumor suppressor of the *SETD2* gene in GC [85].

In colorectal cancer (CRC), the third most common and second most deadly cancer worldwide, SETD2 has also been shown to play a suppressive role [86]. In their clinical and histopathological study of SETD2 in colorectal cancer, Bushara *et al*. thus reported that *SETD2*-mutated CRC affects the proximal and distal colon with equal frequency and that most of the mutations are missense without any “hotspot” mutation sites within the *SETD2* gene. Moreover, the loss of *SETD2* is associated with the loss of H3K36 trimethylation and the aberrant cytoplasmic/nuclear beta-catenin expression [87]. These findings are in line with the study of Yuan *et al*. which evidenced that SETD2 counteracts Wnt signaling and that its inactivation promotes intestinal tumorigenesis in mouse models of CRC [88].

However, it is also reported that SETD2 was not required for intestinal homeostasis under steady state; even if, upon irradiation, the authors demonstrated that genetic inactivation of *SETD2* in mouse intestinal epithelium facilitated the self-renewal of intestinal stem/progenitor cells as well as tissue regeneration [88]. These data should be discussed in the light of the results of Liu *et al*. where SETD2 is described as a modulator of oxidative stress that attenuates colonic inflammation and tumorigenesis in mice. Indeed, this study shows that *SETD2* depletion triggers an excess of reactive oxygen species (ROS) by directly down-regulating antioxidant genes, leading to defects in barrier integrity and subsequently inflammatory damage. These data hence underline a role for SETD2 in the regulation of epithelial homeostasis of the intestinal mucosal barrier [89].

In pancreatic cancer, the expression of SETD2 is significantly decreased compared with normal tissues in around 10 % of the patients [90]*.* One hypothesis about the role of SETD2 in the initiation and metastasis in pancreatic cancer would be on implication in cell metabolism. Indeed, a loss of *SETD2* has been shown to enhance glycolysis addiction through the upregulation of glucose transporter 1 (GLUT1) and to impair nucleoside synthesis by direct downregulation of the transcriptional level of transketolase in the pentose phosphate pathway. *SETD2* deficiency in pancreatic cancer thus reprograms glycolytic metabolism to compensate for insufficient nucleoside synthesis [91].

##### Lung cancer

In lung cancer, inactivation of *SETD2* and subsequent loss of H3K36me3 led to an acceleration of progression of both early and late-stage tumors. SETD2 is also inactivated at a higher frequency in metastatic sites compared with primary sites, which is linked to poor prognosis [92,93]. As previously discussed, in lung adenocarcinoma a loss of *SETD2* favors cell proliferation, migration, invasion and EMT through regulation of the STAT1–IL-8 signaling pathway [57]. Moreover, SETD2 has also been involved in a cisplatin-resistance mechanism in lung adenocarcinoma [94].

##### Breast Cancer

*SETD2* has been described to be mutated in all subtypes of breast cancer up to 2.62 %, and notably SETD2 mutation incidence in triple-negative breast cancer is 1.2 %. The level of SETD2 mRNA is shown to be significantly lower in oncogenic tissues and it further decreases with increasing tumor stage. These observations are consistent with a possible tumor suppressor function of SETD2 in breast cancer and SETD2 has been suggested to play a role as a prognostic indicator in human breast cancer since it is associated with poor prognosis [95,96].

One of the first hypotheses that have been proposed for SETD2-dependent development of breast tumors relies on telomerase regulation through human telomerase reverse transcriptase (hTERT). Indeed, SETD2 has been thought to interact with hTERT through histone methylation of the promoter region in order to regulate telomerase activity [97]*.* However, a study by Linne *et al*. in 2017 showed that SETD2 expression was not associated with repression of hTERT transcription [98].

More recently, circular RNA circ_SETD2, produced by reverse splicing of *SETD2* gene, has been shown to repress breast cancer progression through the regulation of SCUBE2 (signal peptide-CUB-epidermal growth factor-like domain-containing 2) via competitively binding to miR-155-5p [99].

##### Gliomas

H3G34R/V mutations frequently occur in histone H3.3 tail and are correlated with reduced H3K36 methylation since they alter the binding of the target lysine subunit. As described above, the H3G34 is essential for the binding of the substrate as it is inserted into a narrow channel that doesn't allow the binding of H3G34R/V mutated histone. SETD2 mutations are more often identified, but not exclusively, in pediatric and young adult high grade gliomas of the cerebral hemispheres [100,101]. Moreover, *SETD2* mutations may also be associated with other alterations such as TP53 or mutations in growth factor pathways such as EGFR to promote tumor development [100].

### Resistance to therapy

As previously discussed, *SETD2* is frequently mutated in AML, MLL and relapsed acute leukemia. A recent study in mice harboring two loss-of-function (LOF) *SETD2* mutation alleles (SETD2F2478L and SETD2Ex6-KO) revealed that *SETD2* mutations lead to a decreased of S and G2/M checkpoint activity on AML cells hence enabling the cells to escape from massive DNA damage-induced S-phase cell cycle arrest and cell death [102]*.* These observations are in line with the results of Mar *et al*., that indicate that in isogenic leukemia cell lines and in murine leukemia *SETD2* mutations led to resistance to DNA-damaging agents, cytarabine (Cytarabine), 6-thioguanine, doxorubicin, and etoposide (Etoposide), but not to a non–DNA damaging agent, L-asparaginase [103,104]. These results differ from those of Zhou *et al*., demonstrating that *SETD2* deficient mice were more sensitive to 5-fluorouracil (5-FU). Indeed, as they worked on Hematopoietic Stem Cells (HSCs) the authors showed that *SETD2* deficient HSCs had a markedly reduced G0 fraction and increased entries into G1 and S/G2/M phases of the cell cycle. Furthermore, these cells also exhibited increased incorporation of BrdU into the DNA, indicative of an accumulation of cycling cells [52].

However, as resistance to all kinds of therapies is one of the main critical barriers to effective cancer treatment, several studies have been conducted to determine the role of SETD2 in cell resistance. For instance, an acquired missense mutation in *SETD2* (p.T1171K) has been associated with cisplatin resistance in non-small cell lung cancer [94]. In addition, it has been shown that cancer-associated mutations in *SETD2* also concern resistance to chemical inhibitors such as Sunitinib, a tyrosine kinase inhibitor commonly used in targeted therapeutics for the treatment of metastatic RCC [105].

Despite these examples of resistance to therapies, there are ways to overcome SETD2-associated drug resistance. For instance, in leukemia where alteration of SETD2 induces a resistance to chemotherapy, treatment with JIB-04, an inhibitor of the H3K9/36me3 demethylase KDM4A, restored H3K36me3 levels and chemotherapy sensitivity *in vitro* and *in vivo* [104]. In ccRCC, where the survival rate is no >11 % in aggressive or metastasized tumors, it has been demonstrated that SETD2 mutants are sensitive *in vitro* and *in vivo* to DNA hypomethylating agent 5-aza-2′-deoxycytidine (DAC) treatment. Indeed, following DAC treatment in a *SETD2*-loss context, mis-splicing events such as exon inclusions or extensions appear to trigger viral mimicry response [76].

## Conclusions and future perspectives

SETD2 is now well-recognized as a pivotal player in cancer. Although the implication of SETD2 in malignant processes are likely to rely mainly on H3K36me3-dependent mechanisms, the recent identification on non-histone substrates involved in cytoskeletal remodelling or cell signaling suggest that other SETD2-dependent biological processes could be at play. In addition, while genetic alterations of *SETD2* in cancer are mainly considered as loss-of-function mutations, it is likely that "gain of functions" mutations of the enzyme may exist in cancer as reported for other H3K36 methyltransferases such as NSD1 and NSD2 [106,107]. The observation of opposed effects of heterozygous *versus* homozygous loss of *SETD2* also underlines the complex role of the enzyme in cancer developments [69]. Interestingly, recent studies based on synthetic lethal interactions between SETD2 and other molecular players such as PI3K (phosphoinositide 3-kinase) [108] or PRC2 (Polycomb repressive complex 2)-dependant H3K27 methylation [109] offers new potential therapeutic strategies. Importantly, a growing number of studies suggest functional cross-talks between SETD2 and its mutated forms and other geneticaly altered cancer-associated proteins. For instance, "cooperation" of SETD2 mutants with chromosomal translocations, such as MLL-fusions proteins, has been reported to contribute to the initiation and maintenance of leukemias with MLL translocations [36]. These "oncogenic cross talk" have been further underlined in studies of MLL-fusion expressing leukemias in which these malignant cells were strongly dependent on SETD2 for survival and proliferation [69,110]. More recently, cooperation of SETD2 and NPM1 mutations in leukemogenesis has been reported for NPM1-AML cells but not in MLL-rearranged AML [111]. Alhtough these studies were mainly conducted on hematopoietic malignancies, they support a "context"-dependent role of SETD2 in cancer that need to be better adressed and expanded. Interestingly, very recent studies have shown association of SETD2 mutations in lung cancer with specific genomic alterations affecting genes such as BRAF or EGFR [112]. SETD2 mutation was further associated with longer immunotherapy response pointing out SETD2 as a promising biomarker of immunotherapy response [112]. Taken together, these studies underline that the understanding of molecular and/or functional cross-talks between SETD2 and other cancer-associated proteins is key to decipher the role of SETD2 in cancer and to achieve specific therapies. In line with this, the development of SETD2 modulator, notably SETD2 inhibitors, is of prime importance but remains limited. Indeed, until now, the only known small molecule inhibitor of SETD2 was sinefungin, a natural compound isolated from bacteria of the genus Streptomyces [113]. With the growing interest for SETD2 as a drug target, several drug discovery projects have recently been undertaken and new synthetic inhibiting compounds have been discovered [114,115]. For instance, by focusing on exploration of the structure-activity relationship Alford *et al.* synthetized a new inhibitor named EZM0414 [116]. This compound has been shown to be a selective and potent inhibitor of SETD2 with robust pharmocodynamic in a mouse xenograft model. This inhibitor is currently in clinical trial.

## Funding

This work was supported by running grants from Université Paris Cité, Centre National de la Recherche Scientifique (CNRS) and Agence Nationale de la Recherche (ANR-22-CE110002-01).

## CRediT authorship contribution statement

**Christina Michail:** Writing – review & editing, Writing – original draft. **Fernando Rodrigues Lima:** Writing – review & editing, Project administration, Funding acquisition. **Mireille Viguier:** Writing – review & editing, Writing – original draft, Project administration, Conceptualization. **Frédérique Deshayes:** Writing – review & editing, Writing – original draft, Project administration, Conceptualization.

## Declaration of competing interest

The authors declare that they have no known competing financial interests or personal relationships that could have appeared to influence the work reported in this paper.
